# Cicatricial Ectropion Secondary to Psoriatic Arthritis

**DOI:** 10.1155/2015/315465

**Published:** 2015-02-24

**Authors:** Carolina P. B. Gracitelli, Tammy Hentona Osaki, Natalia Yumi Valdrighi, Giovanni André Pires Viana, Midori Hentona Osaki

**Affiliations:** ^1^Ophthalmic Plastic Surgery Division, Department of Ophthalmology, Federal University of São Paulo, Rua Botucatu, 821 Vila Clementino, 04023-062 São Paulo, SP, Brazil; ^2^Ver Mais Oftalmologia, 07750-000 Cajamar, SP, Brazil

## Abstract

Ectropion is characterized by the eversion of the eyelid margin and the consequent exposure of the conjunctiva and cornea. The shortening of the anterior lamella of the lid causes cicatricial ectropion. We described a case of skin pathology causing cicatricial ectropion. The case is about a 68-year-old woman with a 2-year history of psoriatic arthritis. She complained of eyelid tearing and redness for two years. Due to the psoriasis, she presented a very dry skin, also in the periocular region, resulting in cicatricial ectropion. A skin graft was indicated to correct the eyelid malposition. Careful investigation should be performed in patients who have a skin disease that can lead to cicatricial ectropion.

## 1. Introduction

Ectropion is a frequent cause of eyelid malposition. It is characterized by the eversion of the margin and exposure of the cornea and conjunctiva. It is classified as congenital (primary and secondary) or acquired. Within the acquired subtype, ectropion can be involutional, paralytic, mechanical, or cicatricial [1].

Cicatricial ectropion occurs when any factor shortens the anterior lamella of the lid. Such factors include congenital malformations, trauma, burns, scarring skin tumors, allergies, medications, surgeries such as blepharoplasty, skin diseases, or involutional changes that result in the loss of skin elasticity [1]. Although there are some cases in the literature describing cicatricial ectropion due to skin disease, some of these pathologies are poorly described and poorly diagnosed by general physicians. There are few cases in the literature reporting dermatological conditions such as psoriatic arthritis leading to eyelid pathologies [2].

In patients with psoriatic arthritis, many extracutaneous manifestations can produce common ocular complications. Signs and symptoms of ocular psoriasis could be subtle and overlooked. The eyelids can be affected with blepharitis, chronic irritation, cicatricial ectropion, or other less common manifestations [2].

In this case report, we report a case of skin disease leading to cicatricial ectropion. The association of psoriatic arthritis with eyelid diseases has been poorly described in the literature. However, this condition can lead to highly symptomatic cicatricial ectropion, and surgical treatment along with the use of ocular lubricants and ointments is usually needed to relieve patients' symptoms.

## 2. Case Report 

A 68-year-old female was referred to our clinic complaining of progressive ocular irritation with a burning sensation, redness, and tearing in both eyes, though worse in the right eye, for two years. She had been diagnosed with psoriatic arthritis two years prior to her admission to our clinic and she had been using only Acitretina (Acitretin 25 mg/day) without any nonsteroidal anti-inflammatory drug for two years. She was not using any medications for her eye symptoms. She also had systemic hypertension and was in use of captopril 50 mg/day. Her family history was unremarkable.

**Figure 1:**
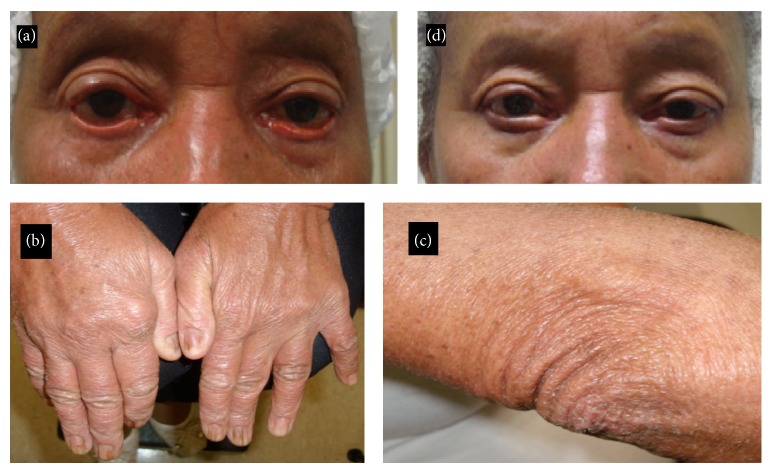
Female patient presenting with psoriatic arthritis. (a) Cicatricial ectropion secondary to psoriatic arthritis; (b) distal interphalangeal arthropathy; (c) elbow skin lesion; (d) improvement of lower eyelid positioning six months after skin graft surgery.

In her ocular examination, the patient's best-corrected visual acuity was 20/20 in both eyes. Her ocular motility and pupil reflex were normal in both eyes. The external examination revealed diffuse dry skin and cicatricial ectropion in both eyes, which was worse in the right eye. The cicatricial ectropion was characterized by lower eyelid eversion and lacrimal punctum eversion (Figure 1(a)). The snap back test and distraction test were unremarkable in both eyes. Biomicroscopy revealed that she had diffuse conjunctival hyperemia, 3+/4+ in the right eye and 2+/4+ in the left eye. The break-up time (BUT) was also decreased (4 seconds). Her intraocular pressure was 14 mmHg in both eyes, without medications, and fundoscopy was normal in both eyes. The rest of the ophthalmological exam was unremarkable. In her complementary exam, some areas (distal interphalangeal joint and elbow) of her skin displayed signs of psoriatic disease (Figures 1(b) and 1(c)).

She underwent a skin graft procedure. Under local anesthesia, an incision was made three millimeters below the eyelid margin beginning at the medial canthus and carried five millimeters beyond the lateral canthus. The inferior aspect of the incision was undermined, creating a defect measuring 2.0 × 4.0 centimeters at its greatest dimensions. This defect was filled with a full-thickness postauricular skin graft sutured in place with interrupted 6-0 nylon. The eyelid was stretched for one week with two double-armed sutures tied to the brow and a tie over bandage was performed.

On the seventh postoperative day the sutures were removed, and after one week the graft became integrated with the original skin. No further ocular symptoms were reported by the patient. An examination disclosed the resolution of the ectropion. The conjunctiva was white and quiet, and the eyelid margins were not inflamed. The BUT was greater than ten seconds. After six months of follow-up, there was no recurrence of the ectropion and she remained asymptomatic and required no ophthalmic medication (Figure 1(d)). There was no recurrence of the ectropion during one year of follow-up.

## 3. Discussion

Our case shows that some skin diseases are responsible for eyelid alterations that can lead to cicatricial ectropion. This pathology may be highly symptomatic and can interfere with the quality of life of these patients. After the surgical treatment, the patient reported relief from her ocular symptoms.

Ectropion can lead to chronic conjunctivitis, keratitis, corneal ulceration, and lagophthalmos, with symptoms of tearing, photophobia, pain, and foreign body sensation [3]. Several cases were reported in the literature showing that skin diseases could lead to cicatricial ectropion [3–5]. Cruz and colleagues, studying the relationship between cornea damage and cicatricial ectropion in eight patients with lamellar ichthyosis, found that all of them had cicatricial lagophthalmos [3]. Another skin disease reported to be correlated with eyelid involvement is paracoccidioidomycosis [4]. Cruz and colleagues reported that 2.5% of their 439 patients with paracoccidioidomycosis experienced cicatricial changes that induced eyelid malpositions such as entropion or ectropion [4]. Discoid lupus erythematosus [5] and cutaneous T-cell lymphoma [6] are other cutaneous diseases that have been reported to result in cicatricial ectropion.

There is little information in the literature about the relationship between psoriatic arthritis and eyelid disorders. A review of the literature by Rehal et al. [2] showed that the ophthalmic complications of psoriasis are numerous and affect several parts of the eye but may be easily missed. Although psoriasis affects 1 to 3% of the adults with multiple extracutaneous manifestations [7], the rate of ocular manifestations is poorly defined. The disease can negatively affect the quality of life of these patients [7, 8]. Among these patients, the ocular manifestations are more common in men and are almost always preceded by cutaneous findings [8]. The mechanisms of such ocular manifestations include the direct involvement of psoriatic plaques or a psoriasis-associated autoimmune process; ocular manifestations often occur during the period of disease exacerbation [8].

Psoriasis can affect the eyelids via different mechanisms. Chronic blepharitis can lead to ectropion, madarosis, trichiasis, loss of lid tissue, and even low visual acuity [8]. Disturbance in the tear film was also reported in these patients [8]. In our case, the patient presented with cicatricial ectropion without madarosis and trichiasis. The tear film was also damaged by the cicatricial ectropion and could be assessed by the BUT exam.

Surgical correction of the ectropion is usually required for symptomatic relief. In our case, we performed a skin graft to correct the anterior lamella defect. Conservative treatment such as steroid ointments, methylcellulose eye drops, and humidified air have been reported as alternative treatments [3, 4]. However, these are not effective, particularly in highly symptomatic patients. Surgical management with a graft or flap is more effective and is indicated if there is shortage of skin [1]. It requires lengthening the anterior lamella and tightening the lower lid [1]. Skin grafts may be obtained from the upper lid, retroauricular region, supraclavicular area, or inner aspects of the upper arm [1]. Recurrent ectropion may occur, but repeated operations appear to have equal success [1].

In general, ocular symptoms in patients with skin diseases are poorly investigated by general physicians. Usually, only patients with advanced symptoms are referred for an ophthalmological evaluation. If the patient could be diagnosed earlier, ocular complications and the negative impact in the quality of life of these patients could be reduced.

In summary, careful investigation should be performed in patients who have a skin disease that can lead to cicatricial ectropion. Mild ocular symptoms such as tearing or photophobia should be the first symptoms alerting the general physician of the need to refer these patients to the ophthalmologist.

## References

[1] de Menezes Bedran E. G., Correia Pereira M. V., Bernardes T. F. (2010). Ectropion. *Seminars in Ophthalmology*.

[2] Rehal B., Modjtahedi B. S., Morse L. S., Schwab I. R., Maibach H. I. (2011). Ocular psoriasis. *Journal of the American Academy of Dermatology*.

[3] Cruz A. A. V., Menezes F. A. H., Chaves R., Coelho R. O., Velasco E. F., Kikuta H. (2000). Eyelid abnormalities in lamellar ichthyoses. *Ophthalmology*.

[4] Cruz A. A. V., Zenha F., Silva J. T., Martinez R. (2004). Eyelid involvement in paracoccidioidomycosis. *Ophthalmic Plastic and Reconstructive Surgery*.

[5] Kopsachilis N., Tsaousis K. T., Tourtas T., Tsinopoulos I. T. (2013). Severe chronic blepharitis and scarring ectropion associated with discoid lupus erythematosus. *Clinical and Experimental Optometry*.

[6] Cook B. E. Jr., Bartley G. B., Pittelkow M. R. (1998). Ophthalmic abnormalities in patients with cutaneous T-cell lymphoma. *Transactions of the American Ophthalmological Society*.

[7] Zachariae H. (1994). Pathologic findings in internal organs in psoriasis. *International Journal of Dermatology*.

[8] Kaldeck R. (1953). Ocular Psoriasis; clinical review of eleven cases and some comments on treatment. *AMA Archives of Dermatology and Syphilology*.

